# Incorporating local ancestry improves identification of ancestry-associated methylation signatures and meQTLs in African Americans

**DOI:** 10.1038/s42003-022-03353-5

**Published:** 2022-04-29

**Authors:** Boyang Li, Bradley E. Aouizerat, Youshu Cheng, Kathryn Anastos, Amy C. Justice, Hongyu Zhao, Ke Xu

**Affiliations:** 1 Department of Biostatistics, School of Public Health, Yale University, New Haven, CT United States; 2 VA Connecticut Healthcare System, US Department of Veterans Affairs, West Haven, CT United States; 3 Bluestone Center for Clinical Research, New York University, New York, NY United States; 4 Department of Oral and Maxillofacial Surgery, New York University, New York, NY United States; 5 Division of General Internal Medicine, Albert Einstein College of Medicine, Montefiore Health System, Bronx, NY United States; 6 Department of Health Policy and Management, Yale University, New Haven, CT United States; 7 Department of Psychiatry, School of Medicine, Yale University, New Haven, CT United States

**Keywords:** DNA methylation, Statistical methods

## Abstract

Here we report three epigenome-wide association studies (EWAS) of DNA methylation on self-reported race, global genetic ancestry, and local genetic ancestry in admixed Americans from three sets of samples, including internal and external replications (*N*_total _= 1224). Our EWAS on local ancestry (LA) identified the largest number of ancestry-associated DNA methylation sites and also featured the highest replication rate. Furthermore, by incorporating ancestry origins of genetic variations, we identified 36 methylation quantitative trait loci (meQTL) clumps for LA-associated CpGs that cannot be captured by a model that assumes identical genetic effects across ancestry origins. Lead SNPs at 152 meQTL clumps had significantly different genetic effects in the context of an African or European ancestry background. Local ancestry information enables superior capture of ancestry-associated methylation signatures and identification of ancestry-specific genetic effects on DNA methylation. These findings highlight the importance of incorporating local ancestry for EWAS in admixed samples from multi-ancestry cohorts.

## Abstract (web summary)

A joint analysis of the VACS and WIHS multi-ethnic cohorts highlights the value in using local ancestry (over global ancestry or self-reported race) in evaluating ancestry-associated DNA methylation signatures.

## Introduction

Differences in DNA methylation across ancestral populations have been observed in different tissues, across health status, and over the life course^1–3^. Early studies identified population differences in DNA methylation at genes of interest in tumor tissues for multiple cancers including breast cancer, non-small cell lung cancer, prostate cancer, and colorectal cancer^4–8^. In noncancerous cells, epigenome-wide association studies (EWAS) have identified thousands of ancestry-associated methylation biomarkers across diverse populations^2,3,9,10^. In neonatal cord blood samples, methylation of over 3000 CpGs showed significant differences between African American and European descent newborns^1,11^. In adult DNA samples from peripheral blood, African American women tend to have overall lower methylation levels when compared with women of European or Hispanic ancestry^12^. Through the analysis of family trio data, 8475 CpG sites in lymphoblastoid cell lines showed different methylation levels between family trios with Northern European ancestry and those with West African ancestry^2^. However, these studies used self-reported race and ethnicity, which are social constructs and typically reflect a complex set of biological and non-biological exposures. Moreover, employing self-reported race or ethnicity may be a low-precision proxy of genetic heterogeneity within each group, particularly in admixed populations, including African Americans and Hispanic Americans.

Ancestral alleles can be estimated for admixed individuals by comparing their genetic data to reference samples collected from individuals from geographically and/or historically anchored ancestry backgrounds. Genetic admixture can be further classified into global ancestry (GA) (by considering markers over the entire genome and deriving an average estimate of ancestry) and local ancestry (LA) (by considering markers over a small segment of the genome and deriving a most probable estimate of ancestry for that segment) components. Methods have been developed to infer population structures for methylation analysis^13,14^. Rahmani et al. developed EPISTRUCTURE, a GA inference approach that identifies DNA methylation signatures associated with nearby genetic variants in reference samples in which both methylation and genotype data are available^14^. Principal components (PCs) of the identified methylation signatures are then computed and shown to be correlated with genotype PCs and thus can be used as proxies to capture population structure^14^. Recently, an EWAS on the GA components identified 194 ancestry-associated methylation sites among individuals with diverse Hispanic origins^15^. Although GA inference provides estimated ancestry origin at the individual level, it is unable to capture the localized admixture heterogeneity across genomic regions that can differ among individuals from admixed groups. LA inference addresses the limitation of GA inference by iteratively estimating the ancestry origin of segments of the genome. It  accommodates the fact that admixture is the result of inheriting segments of the genome which generally shows significant interindividual variability and thus enables fine mapping of substructure for each individual.

The development of computational approaches to infer LA using genotype information has permitted inference of ancestry origin at the haplotype level resolution and capture of the admixture across genomes for admixed individuals^16–19^. Multiple studies have shown that local ancestry is linked to global ancestry in the sense that the average of local ancestry estimates approximated global ancestry estimates^20–22^. RFMix adopted a discriminative approach that simultaneously models the reference panel and admixed samples^19^ and demonstrated accuracy of ancestry inference in diverse simulation settings^23,24^. LA inference has been incorporated in the identification of genetic associations for a number of complex phenotypes and improved admixture mapping of population-specific signals^24–29^. Genetic association studies integrating local ancestry have facilitated the estimation of population-specific genetic effects and detected additional signals that may have been missed by overlooking the ancestry background of genetic variations^24,28^.

Accounting for LA in epigenetic studies of DNA methylation is nascent. Galanter et al. showed that the effects of GA on DNA methylation were partially attributed to cis-acting LA and estimated that LA explained a median of 10% of the variations in GA-associated DNA methylation^30^. Rawlik et al. investigated tissue-specific effects of LA on DNA methylation, identifying 552 CpG sites in whole blood and 337 CpG sites in colorectal tissue from Colombian individuals^21^. Although LA analysis of DNA methylation has the potential to capture heterogeneity in genetic admixture with high resolution, there remain few exemplars incorporating LA into EWAS, and no study could be identified that empirically compares the impact of how ancestry is estimated (i.e., self-reported race, GA, and LA) on EWAS findings.

In this study, we investigate DNA methylation in blood associated with different ancestry variables (self-reported race, GA, and LA), using samples from the Veterans Aging Cohort Study (VACS)^31^ and the Women’s Interagency HIV Study (WIHS)^32^ where both genotype and methylation data are available. We characterized ancestry-associated DNA methylation by performing enrichment analyses on multiple genomic features and estimating the SNP-based heritability for the identified signals. Furthermore, we incorporated LA in the identification of methylation quantitative trait loci (meQTL) and identified significant differences in the genetic effects based on an approach accounting for ancestry origins. Our results demonstrate the utility of LA inference in the characterization of genetic admixture and the identification of ancestry-associated methylation signatures. Our findings have important implications for the conduct of epigenetic studies in admixed populations and for the impact of how ancestry is incorporated into epigenetic studies of DNA methylation.

## Results

We studied DNA methylation and genetic data among African American (AA) and European American (EA) participants from the VACS (*N* = 994) and WIHS (*N* = 230). The VACS samples were randomly divided into two groups and DNA methylation data were collected separately using the Illumina HumanMethylation 450 K (HM450 K) and MethylationEPIC (EPIC) beadchips at different processing times. Even though the two arrays produced highly correlated methylation levels, the array-specific batch effects may confound the EWAS associations if combined and analyzed together. To investigate the batch effects induced by HM450K and EPIC arrays, we analyzed DNA methylation at 408,583 common probes shared by two arrays among 176 samples that were measured with both arrays. Of note, these 176 samples were only included in the discovery group and excluded from the internal replication cohort later in the EWAS and meQTL identifications. Using principal component analysis (PCA), we found that the top 3 PCs explained more than 50% of the methylation variance (Supplementary Fig.1a) and HM450K and EPIC methylation showed distinct clusters in the same samples (Supplementary Fig. 1b). The separation between arrays indicated that even for the same individuals at the shared probes, the measured methylation can be different between the two arrays due to batch effects. Thus we designated the subgroup of samples measured using the HM450K as the primary discovery (*N*_AA_ = 478, *N*_EA_ = 49) group and the subgroup measured using the EPIC as the internal replication (*N*_AA_ = 422, *N*_EA_ = 45) group. The DNA methylation data in WIHS samples were measured using the EPIC and served as an external replication cohort (*N*_AA_ = 131, *N*_EA_ = 99). Demographic and clinical characteristics for the three groups are summarized in Table 1.

**Table 1:** Demographic and clinical characteristics of participants in the Veterans Aging Cohort Study (VACS) and Women’s Interagency HIV Study (WIHS).

Race	(*N*)	VACS discovery group (Illumina HM450K)			VACS internal replication group (Illumina EPIC)			WIHS external replication group (Illumina EPIC)		
		AA (*N* = 478)	EA (*N* = 49)	*P*-value	AA (*N* = 422)	EA (*N* = 45)	*P*-value	AA (*N* = 131)	EA (*N* = 99)	*P*-value
Sex-male	(%)	100%	100%	N/A	100%	100%	N/A	0	0	N/A
HIV-positive	(%)	100%	100%	N/A	100%	100%	N/A	64.12%	53.54%	0.14
Age	(years)	49.44 ± 7.24	49.04 ± 9.39	0.78	47.88 ± 8.04	48.22 ± 6.87	0.76	44.04 ± 9.64	44.69 ± 9.23	0.61
Adherence to medication	(%)	78.09%	81.25%	0.75	75.90%	80.00%	0.67	N/A	N/A	N/A
Viral load	(log10)	2.72 ± 1.24	2.53 ± 1.21	0.29	2.72 ± 1.24	2.51 ± 1.24	0.29	2.08 ± 0.54	2.09 ± 0.72	0.92
Smoking-smokers	(%)	61.23%	59.18%	0.90	56.76%	68.89%	0.16	75.57%	72.73%	0.74
Alcohol-hazardous drinking	(%)	N/A	N/A	N/A	N/A	N/A	N/A	39.23%	50.50%	0.12
PEth	(log10)	1.61 ± 2.32	1.05 ± 2.21	0.11	1.64 ± 2.28	0.98 ± 2.34	0.10	N/A	N/A	N/A
White blood cells		5.18 ± 1.95	5.80 ± 1.91	0.04	5.22 ± 1.81	5.99 ± 1.95	0.02	N/A	N/A	N/A
CD4 T cells		0.05 ± 0.05	0.05 ± 0.06	0.80	0.07 ± 0.06	0.07 ± 0.05	0.44	0.30 ± 0.12	0.33 ± 0.13	0.06
CD8 T cells		0.18 ± 0.08	0.16 ± 0.07	0.05	0.17 ± 0.08	0.14 ± 0.07	0.07	0.24 ± 0.10	0.21 ± 0.10	8.36E-03
Granulocytes		0.52 ± 0.13	0.56 ± 0.10	4.84E-03	0.50 ± 0.12	0.53 ± 0.09	0.03	0.01 ± 0.04	0.01 ± 0.03	0.92
Natural Killer cells		0.08 ± 0.06	0.08 ± 0.05	0.81	0.08 ± 0.05	0.09 ± 0.05	0.21	0.15 ± 0.08	0.15 ± 0.08	0.93
B cells		0.09 ± 0.05	0.07 ± 0.04	0.03	0.11 ± 0.05	0.09 ± 0.04	5.33E-03	0.16 ± 0.07	0.15 ± 0.08	0.09
Monocytes		0.12 ± 0.04	0.11 ± 0.05	0.07	0.10 ± 0.04	0.11 ± 0.04	0.83	0.15 ± 0.07	0.16 ± 0.08	0.11

### Comparison of self-reported race, global genetic ancestry, and local genetic ancestry

**Fig. 1:**
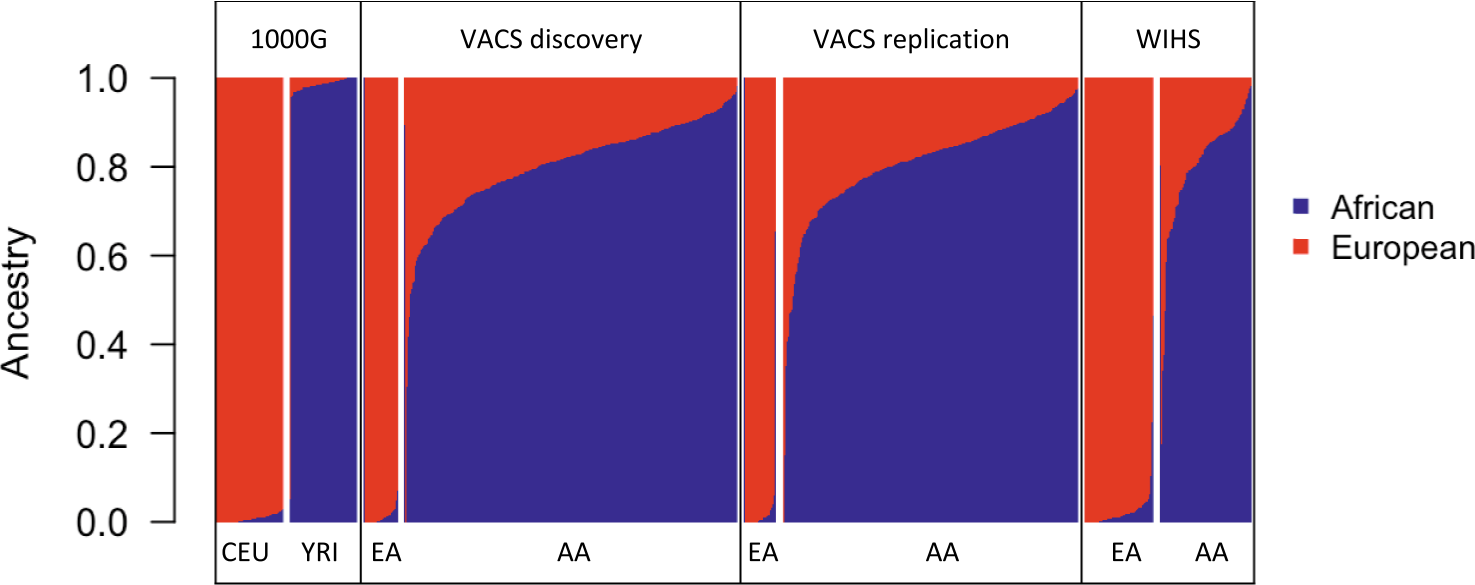
Global ancestry estimated using ADMIXTURE for African Americans and European Americans in the Veterans Aging Cohort Study (VACS) discovery group, VACS internal replication group, and the Women’s Interagency HIV Study (WIHS) external replication group. Yoruba in Ibadan, Nigeria (YRI) and Utah Residents (CEPH) with Northern and Western European Ancestry (CEU) samples from the 1000 Genomes Project are used as the African and European reference panels.

We estimated the African and European ancestry compositions from genotype data for self-reported AAs and EAs in the groups (methods). The individual-level global African (AFR%) and European (EUR%) ancestry proportions were estimated using the 1000 Genomes Project as the reference genotype panel. The global genetic ancestry of self-reported EA samples is predominately made up of European ancestry (Fig. 1). Among the 193 genotyped samples from self-reported EA spanning the three groups, 185 samples had a European ancestral proportion (EUR%) greater than 90%, 3 samples had EUR% that ranged from 70 to 90%, 2 samples had EUR% that ranged from 30 to 60%, and 3 samples had EUR% less than 20%. In comparison, the AA samples displayed more admixed genetic ancestry compositions (Fig. 1). Among the 1031 genotyped samples from self-reported AA spanning the three groups, 1027 samples had an African ancestral proportion (AFR%) that ranged from 15 to 100%, 4 samples had AFR% less than 5%. The wide range of genetic ancestry composition among the self-reported AA samples highlights the high degree of diversity in genetic admixture in the self-reported AA population.

**Fig. 2:**
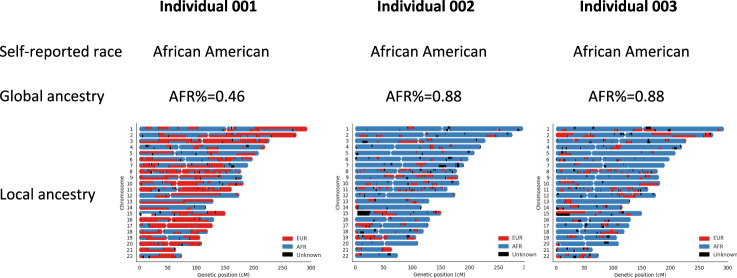
Self-reported race, global ancestry, and local ancestry across 22 chromosomes for 3 self-reported African Americans in the Veterans Aging Cohort Study cohort. Race was extracted from self-reported survey data. Global ancestry was estimated using ADMIXTURE. Local ancestry was estimated using RFMix. The horizontal axis represents genomic coordinates in centimorgans and the vertical axis represents 22 chromosomes, each has two strands, and the color indicates local ancestry designation inferred from RFMix. Yoruba in Ibadan, Nigeria (YRI) and Utah Residents (CEPH) with Northern and Western European Ancestry (CEU) samples from the 1000 Genomes Project are used as the African and European reference panels for global and local ancestry estimations.

We further estimated the most probable ancestral origin (African or European) at each locus for all samples (methods). Because our goal was to understand the impact of ancestry on DNA methylation, local ancestry estimates were anchored by measured CpG sites. Local ancestry at each methylation position (CpG) was defined as a weighted average of local ancestry composition based on genetic variants within a flanking region of 1 megabase (Mb) pairs of a CpG site. The weights were inversely proportional to the distance between a given genetic variant to the CpG site. It is worth noting that for AA samples with a comparable global African ancestry proportion, the distribution of African ancestry across 22 chromosomes varied greatly (Fig. 2). We then evaluated the consistency between the global and local ancestry. Similar to previous report^30^, the proportion of genetic loci with local African ancestry (estimated using the average of local ancestry across the genome) was highly correlated with the global African ancestry in VACS samples (Pearson correlation = 0.999, *p*-value < 2e–16)(Supplementary Fig. 2a) and WIHS samples (Pearson correlation = 0.999, *p*-value < 2e–16) (Supplementary Fig. 2b).

In addition to genetic ancestry, the PCA is a widely employed approach to identify global population structure in the samples. The first PC explained 4.5% of genotype variance in the VACS cohort and was highly correlated with the global African ancestry (Pearson correlation = 1, *p*-value < 2e–16) (Supplementary Fig. 2c). Each of the remaining PCs explained less than 0.2% of genotype variance. We observed similarly patterns in WIHS samples. The first PC explained 7.7% of genotype variance and was highly correlated with the global African ancestry (Pearson correlation = 1, *p*-value < 2e–16) (Supplementary Fig. 2d). Each of the remaining PCs explained less than 0.4% of genotype variance. This indicated that the first PC would suffice to distinguish AAs and EAs in the two cohorts and the remaining PCs captured more subtle within-population structure that contributed minimally to the differentiation of the two ancestries (African and European).

### Epigenome-wide association studies identified ancestry-associated DNA methylation

We performed an EWAS on the self-reported race (binary coded: AA as 1, EA as 0) in EA and AA samples in the VACS discovery group. The global and local ancestry-based EWAS were performed in AAs only to pinpoint methylation signatures associated with genetic ancestry. Because the VACS and WIHS cohorts originally focused on persons living with HIV, our EWAS controlled for risk factors that have been associated with differential methylation in the literature. Age, HIV-related covariates (viral load and adherence to medication), smoking status, alcohol use, white blood cell counts, cell type proportions, methylation PCs at control probes, and residual PCs were included as covariates in the association model for all EWASs (methods). We used 1.16e–7 as the epigenome-wide significance cutoff to declare statistically significant associations. The replication significance cutoff was determined by applying Bonferroni correction to the number of signals identified in the discovery group.

**Fig. 3:**
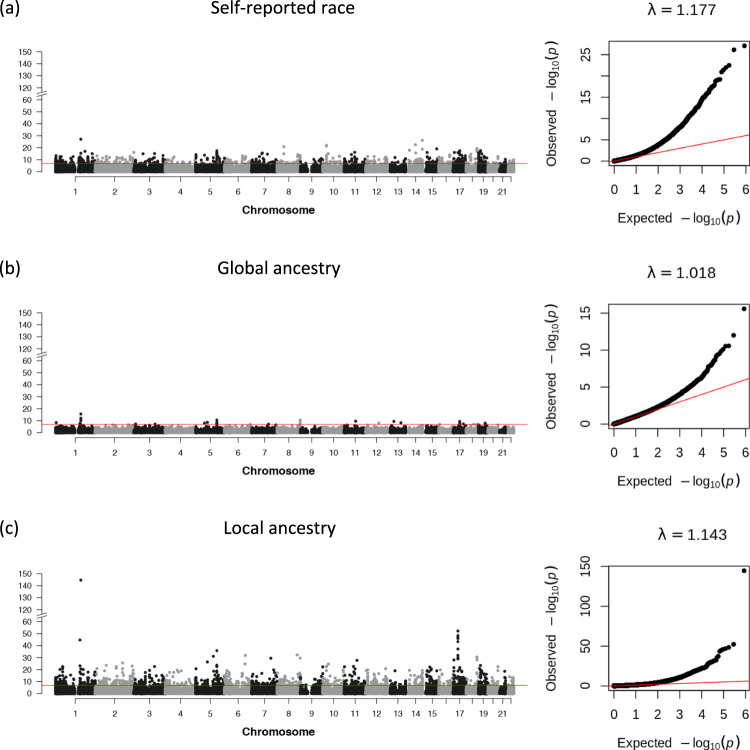
Manhattan and QQ plots for epigenome-wide association study (EWAS) of ancestry variables in the Veterans Aging Cohort Study (VACS) discovery group. The EWAS identified (**a**) 708 CpGs (genomic inflation λ = 1.18) significantly associated with self-reported race (*N* = 527), (**b**) 30 CpGs (genomic inflation λ = 1.02) significantly associated with global ancestry (*N* = 478), and (**c**) 1,284 CpGs (genomic inflation λ = 1.14) significantly associated with local ancestry (*N* = 478), respectively. Self-reported race was extracted from self-reported survey data. Global ancestry was estimated with ADMIXTURE. Local ancestry was estimated using RFMix. Yoruba in Ibadan, Nigeria (YRI) and Utah Residents (CEPH) with Northern and Western European Ancestry (CEU) samples from the 1000 Genomes Project are used as African and European reference panels for global and local ancestry estimations. The vertical axes across three panels are made on the same scale for comparison.

In the VACS discovery group, we identified 708 CpGs (genomic inflation λ = 1.18), 30 CpGs (genomic inflation λ = 1.02), and 1284 CpGs (genomic inflation λ = 1.14) significantly associated with self-reported race (*N* = 527) (Fig. 3a and Supplementary Data 1), GA (*N* = 478) (Fig. 3b and Supplementary Data 2), and LA (*N* = 478) (Fig. 3c and Supplementary Data 3), respectively. The EWAS of LA identified the largest number of ancestry-associated DNA methylation that partially overlapped with those identified for the self-reported race and GA. Specifically, among 708 race-associated CpG sites, 350 (43%) of them overlapped with CpG sites significantly associated with LA. Among 30 GA-associated CpG sites, 15 (50%) of them were also significantly associated with LA. We further compared the coefficient estimates for the overlapped CpG sites. The correlation of estimated effects between LA- and GA-associated CpG sites was 0.985 (*n* = 15 CpG sites, *p*-value = 3.6e–11). The correlation of estimated effects between LA- and race-associated CpG sites was 0.975 (*n* = 350 CpG sites, *p*-value < 2.2e–16). All overlapping CpG sites displayed concordant directions of effects.

The most significant CpG site associated with LA was cg04922029 (*p*-value = 2.2e–145) that mapped to *DARC* on chromosome 1. A higher proportion of local African ancestry around cg04922029 was associated with an increased level of methylation at this CpG site. Specifically, a 25% increase in the local African ancestry proportion was associated with an increased methylation *M*-value of 0.77 conditional on the adjusted covariates. Methylation at *DARC* cg04922029 also showed significant associations with self-reported race (*p*-value = 8.3e–28) and GA (*p*-value = 2.7e–16). African ancestry was consistently associated with increased methylation at cg04922029. Specifically, in the EWAS of self-reported race, AAs had an average increase of 1.9 in the methylation *M*-value than EAs at this CpG site. In the EWAS of GA among AAs, a 25% increase in the global African ancestry was associated with an increased methylation *M*-value of 0.87 at this CpG site. It is noteworthy that Galanter et al. previously reported that hypermethylation at *DARC* cg04922029 was associated with global African ancestry and each 25% increase in the global African ancestry was associated with an increase of 0.98 in the methylation *M*-value^30^.

We performed a sensitivity analysis for LA EWAS accounting for different flanking regions used in the LA definition. By default, the LA at each CpG was defined as a weighted average of local ancestry composition based on genetic variants within 1 Mb flanking region. We compared the significant CpG sites identified for LA using 250 kb, 500 kb, and 1 Mb definition. The three EWAS identified 1279, 1269, and 1284 significant CpG sites, respectively, where 1259 CpG sites were in overlap. Not only did the majority of identified significant CpG sites overlap, the estimated effects were also highly consistent (Pearson correlation>0.99) among different flanking regions (Supplementary Fig. 3). Thus, the LA EWAS associations were relatively robust to different flanking regions used in the LA definition. We proceed with the EWAS results using the 1 Mb definition for LA.

To replicate the significant CpG sites identified in the VACS discovery group, we examined the association of CpG sites for self-reported race, GA, and LA separately in two replication groups. For self-reported race, 312 of 708 (44%) significantly associated methylation sites were replicated in the VACS replication group and 25 (4%) were replicated in the WIHS replication group (*p*-value < 7.06e–5) (Supplementary Data 1). For GA, 14 of 30 (47%) significantly associated methylation sites were replicated in the VACS replication group and 6 (20%) were replicated in the WIHS replication group (*p*-value < 1.67e–3) (Supplementary Data 2). For LA, a total of 771 of 1284 (60%) significantly associated CpGs were replicated with concordant direction of effects in the VACS replication group and 223 (17%) were replicated in the WIHS replication group (*p*-value < 3.89e–5) (Supplementary Data 3). Despite the fact that the LA EWAS had a smaller sample size (after excluding EA samples) than the self-reported race EWAS, we identified more associations for LA in the VACS discovery group with a higher replication rate in both replication groups. Moreover, the estimated effects of the significant LA-associated CpGs were highly correlated between the discovery and the replication groups (Pearson correlation = 0.96 between the VACS discovery and replication groups, Pearson correlation = 0.93 between the VACS discovery group and WIHS replication cohort) (Supplementary Fig. 4). Because the two replication groups were profiled with the EPIC array, we were not able to replicate CpG sites unique to the 450 K array including the most significant LA-associated CpG site cg04922029.

### Downstream analyses characterizing ancestry-associated DNA methylation

**Fig. 4:**
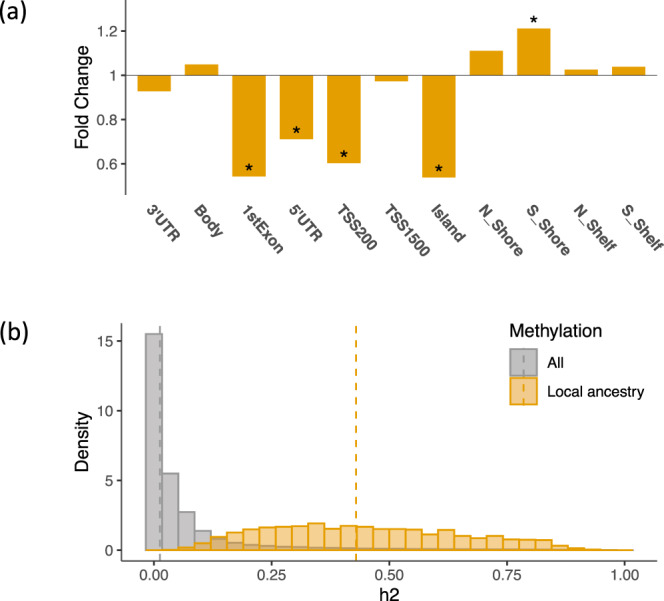
Downstream characterization of the local ancestry-associated DNA methylation. **a** The enrichment or depletion of genomic annotations for the DNA methylation identified in the epigenome-wide association studies (EWAS) of local ancestry. Asterisks (*) indicate significant enrichments or depletions with FDR adjusted *p*-values less than 0.05. **b** The SNP-based heritability estimates of local ancestry associated DNA methylation (mean *h*^2^ = 0.45, median *h*^2^ = 0.43) are considerably higher than the overall heritability (mean *h*^2^ = 0.06, median *h*^2^ = 0.01) estimated from all methylation sites. The SNP-based heritability for each methylation site is estimated using all SNPs in a flanking region of 1 mega basepairs. The source data are provided in Table 2 and Supplementary Data 4.

We performed enrichment analyses using genomic features to characterize the identified DNA methylation associated with LA, GA, and self-reported race, respectively. The CpG sites associated with LA were significantly depleted in the 1st Exon (fold change = 0.54, *p*-value = 7.7e-8), 5′UTR (fold change = 0.71, *p*-value = 1.1e-5), and genic region 200 base pairs (bp) upstream of the transcription start site (also known as TSS200, fold change = 0.60, *p*-value = 2.8e-9) (Table 2 and Fig. 4a). We also examined the CpG positions relative to the CpG island and identified a significant enrichment in regions 0–2 kilobase (kb) downstream of CpG islands (also known as S_Shore, fold change = 1.21, *p*-value = 7.2e-3) and a significant depletion in CpG islands (fold change = 0.54, *p*-value = 1.9e-32)(Table 2 and Fig. 4a). We observed similar significant enrichment in S-Shore (fold change = 1.38, *p*-value = 6.3e-4) and significant depletions in CpG islands (fold change = 0.59, *p*-value = 7.5e-15), 1st Exon (fold change = 0.57, *p*-value = 1.6e-4, 5′UTR (fold change = 0.77, *p*-value = 8.1e-3), and TSS 200 regions (fold change = 0.43, *p*-value = 7.9e-11) for CpG sites associated with self-reported race (Table 2). No significant enrichment or depletion was identified for GA-associated DNA methylation.

**Table 2:** The enrichment or depletion of genomic annotations for the differentially methylated CpG sites identified in the EWAS of local ancestry, global ancestry, and self-reported race.

Genomic Annotation	Local ancestry				Global ancestry				Self-reported race			
	Fold change	*P*-value	FDR	Significance	Fold change	*P*-value	FDR	Significance	Fold change	*P*-value	FDR	Significance
3′UTR	0.93	3.3E–01	4E–01						1.12	2.8E–01	3.1E–01	
Body	1.05	9.7E–02	2E–01		1.01	5.6E–01	5.6E–01		0.91	4.2E–02	7.7E–02	
1stExon	0.54	7.7E–08	3E–07	*	0.82	5.5E–01	5.5E–01		0.57	1.6E–04	6.0E–04	*
5′UTR	0.71	1.1E–05	3E–05	*	0.97	6.1E–01	6.1E–01		0.77	8.1E–03	1.8E–02	*
TSS200	0.60	2.8E–09	2E–08	*	0.77	4.4E–01	4.4E–01		0.43	7.9E–11	4.3E–10	*
TSS1500	0.97	3.4E–01	4E–01		1.70	6.8E–02	6.8E–02		1.00	5.0E–01	5.0E–01	
Island	0.54	1.9E–32	2E–31	*	0.64	1.2E–01	1.2E–01		0.59	7.5E–15	8.2E–14	*
N_Shore	1.11	6.9E–02	1E–01		1.02	5.6E–01	5.6E–01		1.13	1.1E–01	1.5E–01	
S_Shore	1.21	7.2E–03	2E–02	*	1.30	3.7E–01	3.7E–01		1.38	6.3E–04	1.7E–03	*
N_Shelf	1.03	4.3E–01	4E–01		1.33	4.5E–01	4.5E–01		0.87	2.5E–01	3.1E–01	
S_Shelf	1.04	4.0E–01	4E–01						1.29	6.2E–02	9.8E–02	

Asterisks (*) indicate significant enrichments/depletions with FDR adjusted *p*-values less than 0.05.

SNP-based heritability was estimated for DNA methylation associated with LA, GA, and self-reported race, respectively, using SNPs in a 1-Mb flanking region. The average number of SNPs surrounding each CpG was approximately 3000. The heritability of LA-associated methylation (mean *h*^2^ = 0.45, median *h*^2^ = 0.43) was considerably higher than the average methylation heritability across the genome (mean *h*^2^ = 0.06, median *h*^2^ = 0.01) (Fig. 4b and Supplementary Data 4). The methylation heritability of CpG sites associated with self-reported race (mean *h*^2^ = 0.39, median *h*^2^ = 0.37) and GA (mean *h*^2^ = 0.40, median *h*^2^ = 0.41) was slightly lower than that identified for LA-associated methylation but higher than the average methylation heritability across the genome. Huan and Joehanes et al. suggested methylation with heritability greater than 0.1 are depleted in promoters, CpG islands, and TSS200 regions^33^, which is consistent with our findings that the identified methylation with relatively high heritability are depleted in the same regions.

We performed trait enrichment analyses using the EWAS Atlas database to identify traits that have overlapped significant CpG sites with LA, GA, and self-reported race, respectively. The most significantly enriched traits for LA-associated methylation were ancestry (odds ratio = 42.99, *p*-value = 0), childhood stress (odds ratio = 52.59, *p*-value = 5.78e–90), and aging (odds ratio = 3.29, *p*-value = 7.66e–67) (Supplementary Data 5). Similar trait enrichments were identified for DNA methylation associated with self-reported race and GA. For self-reported race, the same three traits were identified as the most significantly enriched traits. The top three most significantly enriched traits for GA were ancestry, childhood stress, and serum immunoglobulin E (IgE) levels.

We also performed gene set enrichment using Gene Ontology (GO) and KEGG pathway annotations. Applying a false discovery rate (FDR) of 0.05, no significant pathway was identified for LA, GA, or self-reported race.

### Identification of local ancestry-associated meQTL

We applied two models to identify pairwise associations between LA-associated methylation and SNPs in each 1 Mb flanking region (Table 3). The first one is a conventional model widely used in the identification of methylation quantitative trait loci (meQTL) that assumes identical effects across ancestral origins of the genotype. The second, the ancestry model, allows SNP genetic effects with an African or European ancestry background to be different on DNA methylation and the significance of the difference in genetic effects can be tested. Local ancestry was adjusted in both models to control for the confounding effects from ancestry background. As many meQTLs are correlated due to linkage disequilibrium (LD), we performed clumping of identified adjacent meQTLs and selected the meQTL with the most significant association as the representative SNP for each meQTL clump (methods). The conventional model identified 43,074 meQTLs (*p*-value < 1.35e–8, F test: conventional vs. null model) that mapped to 1269 meQTL clumps (Supplementary Data 6). The ancestry model allows the genetic effects to be different for SNPs with an African or European ancestry background and it identified 44,613 meQTLs (*p*-value < 1.35e–8, F test: ancestry vs. null model) that mapped to 1268 meQTL clumps (Supplementary Data 7). A total of 1,232 meQTL clumps were identified by both models, 37 meQTL clumps were uniquely identified by the conventional model, and 36 meQTL clumps were uniquely identified by the ancestry model. The *p*-values from the ancestry model for the 37 meQTL clumps identified uniquely using the conventional model approached the significance cutoff for the ancestry model (*p*-values ranged from 1.38e–08 to 9.73e–08). On the other hand, for the 36 meQTL clumps missed by the conventional model, many had *p*-values larger than the nominal significant cutoff of 0.05. It is noteworthy that lead SNPs at 8 of 36 meQTLs clumps had opposite genetic effects in the context of local African or European ancestry background. The conventional model aggregated the genotype counts regardless of the ancestral background and the ancestral effects with opposite directions mutually attenuated, leading to a non-significant result.

**Table 3:** Methylation quantitative trait loci (meQTL) model specifications for the null, conventional, and ancestry models.

Model	Model specification
Null	${{{{{\rm{Methylation}}}}}}=\mathop{\sum }\limits_{i=1}^{p}{b}_{i}\times {{{{{{\rm{covariate}}}}}}}_{i}+{b}_{{LA}}\times {{{{{\rm{LA}}}}}}+\varepsilon$
Conventional	${{{{{\rm{Methylation}}}}}}=\mathop{\sum }\limits_{i=1}^{p}{b}_{i}\times {{{{{{\rm{covariate}}}}}}}_{i}+{b}_{{LA}}\times {{{{{\rm{LA}}}}}}+{b}_{{SNP}}\times {{{{{\rm{SNP}}}}}}+\varepsilon$
Ancestry	${{{{{\rm{Methylation}}}}}}=\mathop{\sum }\limits_{i=1}^{p}{b}_{i}\times {{{{{{\rm{covariate}}}}}}}_{i}+{b}_{{LA}}\times {{{{{\rm{LA}}}}}}+{b}_{{AFR}}\times {{{{{{\rm{SNP}}}}}}}_{{AFR}}+{b}_{{EUR}}\times {{{{{{\rm{SNP}}}}}}}_{{EUR}}+\varepsilon$
	$$=\mathop{\sum }\limits_{i=1}^{p}{b}_{i}\times {{{{{{\rm{covariate}}}}}}}_{i}+{b}_{{LA}}\times {{{{{\rm{LA}}}}}}+\frac{{b}_{{AFR}}\,+\,{b}_{{EUR}}}{2}\left({{{{{{\rm{SNP}}}}}}}_{{AFR}}+{{{{{{\rm{SNP}}}}}}}_{{EUR}}\right)+\frac{{b}_{{AFR}}\,-\,{b}_{{EUR}}}{2}\left({{{{{{\rm{SNP}}}}}}}_{{AFR}}-{{{{{{\rm{SNP}}}}}}}_{{EUR}}\right)+\varepsilon$$
	$$=\mathop{\sum }\limits_{i=1}^{p}{b}_{i}\times {{{{{{\rm{covariate}}}}}}}_{i}+{b}_{{LA}}\times {{{{{\rm{LA}}}}}}+\frac{{b}_{{AFR}}\,+\,{b}_{{EUR}}}{2}\left({{{{{{\rm{SNP}}}}}}}_{{AFR}}+{{{{{{\rm{SNP}}}}}}}_{{EUR}}\right)+\left({b}_{{AFR}}-{b}_{{EUR}}\right)\frac{{{{{{{\rm{SNP}}}}}}}_{{AFR}}\,-\,{{{{{{\rm{SNP}}}}}}}_{{EUR}}}{2}+\varepsilon$$
	$$=\mathop{\sum }\limits_{i=1}^{p}{b}_{i}\times {{{{{{\rm{covariate}}}}}}}_{i}+{b}_{{LA}}\times {{{{{\rm{LA}}}}}}+{b}_{{average}}{{{{{\rm{SNP}}}}}}+{b}_{{diff}}\frac{{{{{{{\rm{SNP}}}}}}}_{{AFR}}\,-\,{{{{{{\rm{SNP}}}}}}}_{{EUR}}}{2}+\varepsilon$$

The null model assumes no genetic effect on the DNA methylation. The conventional model assumes identical effects across ancestral origins of the genotype. The ancestry model allows SNP genetic effects with an African or European ancestral background to be different on the DNA methylation.

**Fig. 5:**
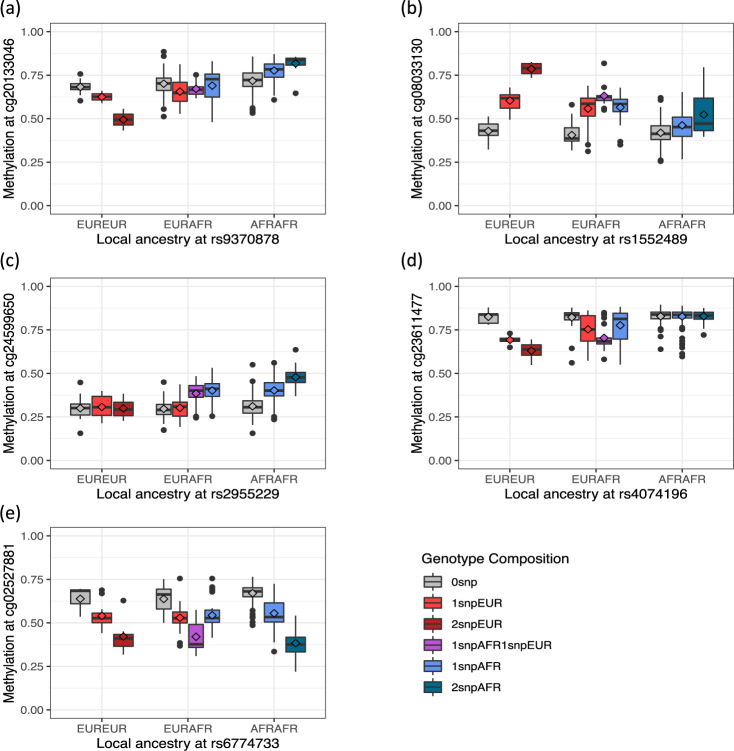
An illustration of four representative patterns of genetic effects by ancestry for the meQTLs identified by the ancestral model. In each panel, the set of boxplots show the distribution of methylation (beta-value) by genotype composition. The lozenge indicates mean of methylation beta-value. **a** The genetic effects on the methylations are opposite for the African and European ancestry. **b** The genetic effects from the two ancestries contribute to the methylation in the same direction but with different effect sizes. **c** The African alleles are associated with differential methylations while the European alleles are not. **d** The European alleles are associated with differential methylations while the African alleles are not. **e** The genetic effects are comparable between ancestries. The box denotes interquartile range (IQR, 25th to 75th percentile) where the central line in the box denotes the median value (50th percentile) and the lozenge denotes the mean value. The upper and lower whisker denotes the largest value within 1.5 times IQR above the 75th percentile and the smallest value within 1.5 times IQR below the 25th percentile, respectively. Values outside of the whisker range are denoted as dots. The source data are provided in Supplementary Data 9–13.

For the identified meQTL, we further evaluated the significance of the difference in SNP effects by local African and European ancestry. Lead SNPs at 152 of 1268 meQTL clumps had significantly different SNP effects by ancestry (*p*-value < 1.12e–6) (Supplementary Data 8). The difference in effect ranged from −2.31 to 5.65. We identified four representative patterns of genetic effects by ancestry for the meQTLs identified by the ancestry model (Fig. 5). The first scenario was that genetic effects on methylation were opposite for the two LA background. For example, the African-ancestry allele at rs9370878 was associated with an increased methylation *M*-value of −0.36 at cg20133046 (located on chromosome 6) whereas the European-ancestry allele was associated with a decreased methylation *M*-value of 0.44 (Fig. 5a and Supplementary Data 9). When aggregated by genotype count, the genetic effects from the two LA background canceled out and the overall genetic effect was not statistically significant. This was the case for 22% of the meQTLs uniquely identified by the ancestry model. In the second scenario, genetic effects from the two LA background contributed to the methylation in the same direction but with different effect sizes. For example, the African-ancestry allele at rs1552489 was associated with an increased methylation *M*-value of 0.32 at cg08033130 (located on chromosome 3 and mapped to *CXCR6*/*FYCO1*). The European-ancestry allele was also associated with cg08033130 hypermethylation but the associated increase was greater (*M*-value of 0.80) (Fig. 5b and Supplementary Data 10). The third scenario was that a genetic effect from only one ancestry was associated with differential methylation levels. For example, the African-ancestry allele at rs2955229 was associated with an increased methylation *M*-value of 0.50 at cg24599650 (located on chromosome 8 and mapped to *RPL8*) while samples with 0, 1, or 2 European-ancestry alleles at rs2955229 had comparable methylations (Fig. 5c and Supplementary Data 11). There are also cases when European-ancestry alleles were associated with differential methylation while the African-ancestry alleles were not (Fig. 5d and Supplementary Data 12). The last scenario was when genetic effects were comparable between the two LA background (Fig. 5e and Supplementary Data 13). In this case, the overall effect was comparable to the ancestry genetic effects and the corresponding meQTL was also identified employing the conventional model.

In replication of the meQTLs, we restricted the evaluation of CpG sites to those that were replicated in the EWAS stage. In the VACS discovery group, 785 lead meQTLs (among which 109 displayed significantly different ancestry effects) were identified for CpG sites that were replicated in the VACS replication group in the EWAS stage. Six hundred forty-nine of 785 (83%) lead meQTLs were replicated (*p*-value < 6.37e–5, *F* test: ancestry vs. null model) in the VACS replication group (Supplementary Data 7) where 58 displayed significantly different ancestry effects (difference ranged from −1.98 to 2.86, *p*-value < 4.59e–4) (Supplementary Data 8). In the VACS discovery group, 185 lead meQTLs (33 with significantly different ancestry effects) were identified for CpG sites that were replicated in the WIHS replication group in the EWAS stage. Eighty-six of 185 (46%) lead meQTLs were replicated (*p*-value < 2.72e–4, *F* test: ancestry vs. null model) in the WIHS replication group (Supplementary Data 7) where 10 displayed significantly different ancestry effects (difference ranged from −1.09 to 1.47, *p*-value < 1.52e–3) (Supplementary Data 8).

## Discussion

In this study, we identified 1,284 LA-associated CpG sites among AAs, with 60% replication rate in an internal replication group and 17% replication rate in an external replication group. We further characterized the LA-associated CpG sites and found that the significant CpG sites were depleted in the functional regions of genes. The LA-associated methylation signatures also showed high SNP-based heritability (mean *h*^2^ = 0.41). Furthermore, by incorporating ancestry origins of genetic variations into the association model and allowing genetic effects to be different by ancestry background, we identified a large number cis-meQTLs for LA-associated CpG sites. Our results demonstrate that LA inference provides a fine mapped population structure in the epigenome. Local ancestry is informative in addressing population admixture for EWAS.

Using the self-reported race as a proxy for ancestral origin is a common practice in recent epigenetic studies. Despite its commonplace collection and convenience, self-reported race and ethnicity are social constructs that fail to accurately reflect the genetic admixture in a population and may result in confounding in EWAS. Our results show that genetically inferred LA is superior to self-reported race in addressing the influence of population admixture on DNA methylation. With ready access to genetic reference data (e.g. the 1000 Genomes Project) and the increasing viability of multi-omics (i.e., genetic, DNA methylome) data in the same sample, it is feasible to infer LA for each individual in a sample and to identify LA-associated DNA methylation signatures. Our EWAS on LA identified more associated DNA methylation sites than EWAS of GA and self-reported race and also featured the highest replication rate for the identified methylation sites in both replication groups. These findings suggest that incorporating LA into EWAS is a superior approach than self-reported race or GA to address the confounding effect from population substructure. We also observed DNA methylation associations that overlapped across EWAS of three different ancestry variables. The overlap in the identified CpG methylation sites is not unexpected given that the ancestry variables overlap in a broad sense that (1) dichotomizing global African ancestry estimates at 10% results in an almost perfect identical agreement with self-reported AAs and EAs; (2) the global ancestry is an average of local ancestry across the genome; and (3) self-reported race and ethnicity is an established, if imprecise, proxy of ancestry in human population genetic association studies.

The genetic contribution to DNA methylation varied widely across CpG sites and the distribution of the SNP-based heritability is heavily right skewed. The downstream analyses of the DNA methylation sites identified across the three approaches to approximate ancestry, demonstrate that ancestry-associated DNA methylation is, on average, highly heritable and significantly depleted in promoter regions (1st Exon, 5′UTR, and TSS200) and CpG islands while moderately enriched in south shores. This agrees with the previous findings of Rawlik et al. that population-specific methylations is depleted in promoter regions and CpG islands while enriched in the intergenic region^21^. Moreover, Huan et al. also showed that heritable DNA methylation sites are depleted in promoter, TSS200, CpG island, and high-CpG dense regions while enriched in enhancer regions^33^. Taken together, these findings suggest that LA-associated DNA methylation is less likely to be located in high-density CpG regions. Based on these findings, we speculate that LA-associated methylation may play an important role in maintaining epigenome stability and warrants functional study.

The characterization of high SNP-based heritability motivated our investigation of the genetic components underlying ancestry-influenced DNA methylation. We incorporated LA in the identification of meQTL. An advantage of this approach is that it allows genetic effects on DNA methylation to be different by ancestry and enables examination of the magnitude and significance of the identified differential effects^34,35^. The ancestry model identified 36 meQTL clumps that were missed by the conventional model where the ancestral origin of genetic variations is not taken into account. More interestingly, the ancestry model identified 97% of the meQTL clumps identified by the conventional model with highly congruent test statistics for meQTL clumps identified by both models and the remaining 3% of the meQTL clumps uniquely identified by the conventional model had test statistics that approached the significance threshold in the ancestry model. However, the opposite does not hold true for the 36 meQTL clumps uniquely identified by the ancestry model (i.e., the test statistics for the same meQTL clumps tested using the conventional model showed little evidence of approaching the significance threshold). Interestingly, 8 of the lead SNPs at these 36 meQTL clumps displayed opposite genetic effects in the two ancestral contexts. As a result, aggregation of genotypes regardless of the ancestral origin can confound statistically significant genetic effects. The fact that lead SNPs at 152 of 1268 of the meQTL clumps displayed significantly different genetic effects based on African and European ancestral contexts indicates that there exists DNA methylation that is affected by the local ancestry-based genetic heterogeneity. This provides additional evidence illustrating the benefit of using LA to identify differentially methylated sites in admixed populations. It also emphasizes the importance of considering the impact of ancestry on the association between genetic variation and DNA methylation and the approach for doing so.

Our study focused on the association between methylation and genetic admixture for AA samples recruited in the HIV studies. LA-associated methylation in the non-HIV population and relevant to Native American, Asian, Hispanics, or other ancestries remain to be evaluated. The number of EA samples was relatively limited in our study. Although the ratio of sample sizes of EA and AA does not bias the estimated effects, the self-reported race EWAS would benefit from an increased EA sample size in terms of smaller standard error. We focused on identifying ancestry-associated DNA methylation measured by HM450K arrays. Additional associations remain to be identified if methylation based on EPIC array were available for both the discovery and replication groups. Our findings are based on bulk (i.e., whole blood, peripheral blood mononuclear cells) DNA methylation signatures that were generated from biospecimens collected from VACS and WIHS participants. Despite that one previous study suggested that population-specific methylation signatures are consistent across tissues^21^, examination of cell type-specific ancestral effects on DNA methylation is warranted. Finally, we only examined cis-meQTL in a 1 Mb flanking region of the ancestry-associated CpG site. Although our meQTL model allowing genetic effects to be different by ancestry can be readily applied to identify trans-meQTL, it would greatly increase the burden of multiple testing and lead to a more stringent significant cutoff. Future study with increased sample size is needed to identify trans-meQTL with ancestry-specific effects. Despite the limitations, we demonstrate the merits of using local ancestry to better capture the impact of admixture and identify ancestry-associated DNA methylation in AA cohorts. We provide a framework for the application of local ancestry estimates to improve the identification and interpretation of DNA methylation signatures for diverse phenotypes. These findings have important implications for the conduct of epigenetic studies in admixed populations.

## Methods

### Study cohort

The Veterans Aging Cohort Study (VACS) and the Women’s Interagency HIV Study (WIHS) are both multi-center, prospective, observational cohort studies^31,32^. The VACS recruited HIV-positive cases and age-, race-, site-matched HIV-negative controls where the majority of participants are men. The study was approved by the committee of the Human Research Subject Protection at Yale University and the Institutional Research Board Committee of the Connecticut Veteran Healthcare System. All VACS subjects provided written consents. In WIHS, all participants are women infected with HIV or at risk for HIV acquisition. Informed consent was provided by all WIHS participants via protocols approved by institutional review committees at each affiliated institution. We studied DNA methylation and genetic data of AA and EA participants from the two cohorts. The VACS samples were randomly divided and measured with HM450K and EPIC arrays. They were separately processed using different platforms and at different processing times. Even though the two arrays produced highly correlated methylation levels, the array-specific batch effects may confound the EWAS associations if combined and analyzed together. Thus we used the HM450K samples as a discovery group (*N*_AA_ = 478, *N*_EA_ = 49) and the EPIC samples as an internal replication group (*N*_AA_ = 422, *N*_EA_ = 45). The WIHS cohort (*N*_AA_ = 131, *N*_EA_ = 99) served as an external replication cohort. Demographic and clinical characteristics for the three groups were summarized in Table 1.

### DNA methylation

The Illumina Infinium HumanMethylation450 BeadChip (HM450K) and the Illumina Infinium MethylationEPIC BeadChip (EPIC) was used for DNA methylation profiling of the VACS discovery group and internal replication cohort, respectively. There was no sample overlap between the two groups. DNA methylation on samples contributed by WIHS participants was profiled using the EPIC array. We followed methods described in Lehne et al. to perform methylation normalization and adjust for potential batch effects^36^. A total of 437,722 CpGs from the HM450K array passed quality control steps and were used in the association analysis for EWAS discovery. A subset of 407,038 CpG sites also covered by the EPIC array were extracted for replication analysis.

### Genotyping, imputation, and quality control

The VACS samples were genotyped using the Illumina HumanOmniExpress Beadchip that targeted approximately 896,000 genetic variants. Imputation was performed with IMUPTE2^37^ and using the 1000 Genomes Project 3 reference panel^38^, resulting in 18 million genetic variants. The WIHS samples were genotyped using the Infinium Omni2.5 BeadChip that targeted approximately 2.4 million genetic variants. Minimac4 was used for imputation^39^ with 1000 Genomes Project 3 as the reference panel^38^, yielding 34 million genetic variants. In both cohorts, we removed insertions and deletions and retained only single nucleotide polymorphisms (SNP) for genetic analyses. We also removed SNPs with minor allele frequency < 0.01, missing rate > 5%, imputation quality *r*^2^  < 0.8, and those that deviated significantly from Hardy–Weinberg equilibrium (*p* < 1e–6). Approximately 4.7 million SNPs passed QC and were used for local ancestry estimation, SNP-based heritability estimation, and meQTL identifications across all three groups.

### Ancestry estimation

We adopted a two-way admixture of African and European ancestry to model ancestry composition for African Americans^24,40,41^. We used Utah residents with Northern and Western European ancestry (CEU) and samples from Yoruba in Ibadan, Nigeria (YRI) recruited in the 1000 Genomes Project as the reference genotype panel for European and African descent for both global and local ancestry inference^38^. Individuals with excessive relatedness from the reference panels were removed from the analysis, resulting in 98 CEU and 97 YRI unrelated reference samples^42^. We took the overlapping SNPs between VACS and 1000 Genomes Project for global and local ancestry estimation. We used ADMIXTURE 1.3.0 to perform global ancestry estimation with the number of ancestral groups set to 2^43^. We pruned genetic variants using PLINK 1.9 with window size set to 250 kilobase (kb) pairs, step size set to 10 kb, and linkage disequilibrium measure r-squared set to 0.05^44^. 40,508 SNPs retained for global ancestry estimation after pruning. The global ancestral compositions were not sensitive to varying parameter choices and resulting number of SNPs. We performed PCA on the same collection of SNPs to visualize population structure based on genotype data. For local ancestry estimation, we first used SHAPEIT2 to phase genotype data for both reference and admixed samples^45^. RFMix 1.5.4 was then used to infer local ancestry of genetic variants from phased samples^19^. Local ancestry at a methylation position (CpG) is defined as a weighted average of local ancestry composition of genetic variants within a flanking region of 1 megabase (Mb) pairs centered around the CpG site. The weights were inversely proportional to the distance between the SNP and a CpG site and then normalized across genetic variants such that the total weights summed to 1. Consequently, the SNPs closer to a CpG site would have greater influence on the local ancestry at that CpG site than SNPs further away.

### Identification of ancestry-associated methylation

We performed EWAS on the self-reported race, GA, and LA, respectively, to identify DNA methylation associated with self-reported and genetic ancestry. We first performed a self-reported race EWAS in EA and AA samples. Next, we restrained the global and local ancestry-based EWAS in AA samples to pinpoint methylation signatures associated with genetic ancestry. The effect of GA or LA on the DNA methylation may be potentially heterogeneous between EA and AA samples. Adjusting for self-reported race as a covariate allowed EA and AA samples to have different baseline DNA methylation. However, it does not capture the potentially heterogeneous effect of GA or LA on DNA methylation between ancestry groups. Moreover, GA and LA usually exhibited little variation in EA samples, resulting in their limited contribution to investigate the effect of genetic ancestry on DNA methylation. Consequently, we excluded a limited number of EA samples and focused on AA samples to investigate the effect of genetic ancestry on the DNA methylation. In the LA EWAS, we further adjusted GA as a covariate. Other covariates included in all three EWAS models were age at baseline, adherence to medication (adherence vs. non-adherence), viral load (log10 scale), smoking status (smoker vs. non-smoker), alcohol use (PEth score measured on log10 scale in VACS and hazardous drinker vs. non-hazardous drinker in WIHS), white blood cell counts, cell-type composition (CD4 T cells, CD8 T cells, Granulocytes, Natural Killer cells, B cells, Monocytes), and first 30 principal components (PCs) of methylation levels measured at control probes.

In each EWAS, we applied a two-stage model to control for technical and biological confounders and reduce EWAS inflation factor following Lehne et al. and Zhang et al^36,46^. First we constructed a model regressing the methylation *M*-value on all covariates (e.g., age, viral load, adherence to medication, smoking status, alcohol use, cell-type composition, control probe PCs) excluding the ancestry variable of interest (self-reported race, GA, or LA) and obtained the PCs of the residuals. The top 5 residual PCs were then adjusted in the second-stage model to reduce the correlation between DNA methylation and test statistic inflation. In the second stage model, we regressed the methylation *M*-value on the ancestry variable of interest, the covariates included in the first model, and the top 5 residual PCs from the first model. The LA EWAS model in VACS was given as an example. Although DNA methylation beta-value has a more intuitive biological interpretation, the heteroscedasticity for highly methylated or unmethylated CpG sites (beta-value close to 1 and 0) is susceptible to violation of linear model assumptions^47^. Thus we used the approximately homoscedastic methylation *M*-value as response variable in both modeling stages for statistical validity. EWAS models for self-reported race and GA in VACS and all replication EWAS models in WIHS were detailed in the Supplementary Note 1. CpG sites with a *p*-value less than the significance cutoff of 1.16e–7 were declared as ancestry-associated DNA methylation biomarkers. The replication significance cutoff was determined by applying Bonferroni correction to the number of signals identified for each ancestry variable in the discovery group, i.e., 7.06e–5 for self-reported race, 1.67e–3 for GA, and 3.89e–5 for LA, respectively. R 4.0.3 was used for implementation of EWAS models and visualizations.

1 $$	{{{{{\rm{Methylation}}}}}}\,{{{{{\rm{M}}}}}}{-}{{{{{\rm{value}}}}}} \sim {{{{{\rm{GA}}}}}}+{{{{{\rm{age}}}}}}+{{{{{\rm{smoker}}}}}}+{{\log }}({{{{{\rm{PEth}}}}}})+{{{{{\rm{ADH}}}}}}+{{\log }}({{{{{\rm{VL}}}}}})\\ 	 +{{{{{\rm{WBC}}}}}}+{{{{{\rm{CD}}}}}}4+{{{{{\rm{CD}}}}}}8+{{{{{\rm{Granulocyte}}}}}}+{{{{{\rm{NK}}}}}}+{{{{{\rm{Bcell}}}}}}+{{{{{\rm{Monocyte}}}}}}\\ 	+{{{{{\rm{PC}}}}}}1{{{{{\rm{ControlProbe}}}}}}+\ldots +{{{{{\rm{PC}}}}}}30{{{{{\rm{ControlProbe}}}}}}$$

2 $$	{{{{{\rm{Methylation}}}}}}\,{{{{{\rm{M}}}}}}{-}{{{{{\rm{value}}}}}} \sim {{{{{\rm{LA}}}}}}+{{{{{\rm{GA}}}}}}+{{{{{\rm{age}}}}}}+{{{{{\rm{smoker}}}}}}+{{\log }}({{{{{\rm{PEth}}}}}})+{{{{{\rm{ADH}}}}}}\\ 	+{{\log }}({{{{{\rm{VL}}}}}})+{{{{{\rm{WBC}}}}}}+{{{{{\rm{CD}}}}}}4+{{{{{\rm{CD}}}}}}8+{{{{{\rm{Granulocyte}}}}}}+{{{{{\rm{NK}}}}}}+{{{{{\rm{Bcell}}}}}}+{{{{{\rm{Monocyte}}}}}}\\ 	 +{{{{{\rm{PC}}}}}}1{{{{{\rm{ControlProbe}}}}}}+\ldots +{{{{{\rm{PC}}}}}}30{{{{{\rm{ControlProbe}}}}}}+{{{{{\rm{PC}}}}}}1{{{{{\rm{Residual}}}}}}+\ldots +{{{{{\rm{PC}}}}}}5{{{{{\rm{Residual}}}}}}$$

### Positional enrichment analyses of DNA methylation sites associated with ancestry

We performed enrichment analyses using genomic features to characterize the identified DNA methylation associated with LA, GA, and self-reported race, respectively. We extracted positional annotations for all probes in the HM450K arrays using the R package IlluminaHumanMethylation450kanno.ilmn12.hg19. We performed enrichment analyses on the RefGene annotations (3′UTR, Body, 1st Exon, 5′UTR, TSS200, and TSS1500) and annotations describing relative position to CpG island (Island, N_Shore, S_Shore, N_Shelf, and S_Shelf)^48^. We first calculated the proportion of probes with a specific annotation, i.e., the annotation coverage, across all the probes. We then calculated the annotation coverage among the significant probes identified in each of the EWAS. The fold change for each annotation is defined as the ratio of the annotation coverage of the identified ancestry-associated DNA methylation sites and the annotation coverage across all DNA methylation sites. A fold change greater than 1 indicates enrichment and a fold change smaller than 1 indicates depletion. The *p*-value associated with the fold change is derived from a hypergeometric test. We simultaneously tested the 11 annotations mentioned above and used the Benjamini–Hochberg false discovery rate (FDR) less than 0.05 as the significance threshold.

### Estimation of DNA methylation heritability

SNP-based heritability was estimated for DNA methylation associated with LA, GA, and self-reported race, respectively, using SNPs in a 1-Mb flanking region. The SNP-based methylation heritability is defined as the proportion of the variation in DNA methylation explained by genetic effects. We used the genome-based restricted maximum likelihood (GREML) method implemented in the genome-wide complex trait analysis (GCTA 1.93.2) tool to estimate the heritability^49^. In the heritability model, genetic effects were modeled as random and the same set of covariates in the EWAS (age at baseline, adherence to medication, viral load, smoking status, alcohol use, cell-type composition, the first 30 PCs of control probe methylations, and the first 5 residual PCs) were used as fixed effects. We compared the distribution of heritability estimates for LA-associated DNA methylation identified in the EWAS to the overall distribution across all measured DNA methylation.

### Trait enrichment analyses of DNA methylation sites associated with ancestry

Trait enrichment analyses were performed by comparing the significant DNA methylation identified for LA, GA, or self-reported race with those reported for other traits in the literature. The EWAS Atlas database was used for this analysis where more than 617,000 associations were documented for 619 traits through curation of 900 publications and EWAS studies^50,51^. Specifically, there were 4 epigenetic studies on ancestry in the database with 11,355 associated CpG sites (https://ngdc.cncb.ac.cn/ewas/browse?traitList=ancestry). EWAS Atlas applied a weighted Fisher’s exact test to compute the co-occurrence probability between ancestry-associated DNA methylation and trait-related DNA methylation reported in the published EWAS. As a result, 87, 23, 82 traits shared at least one significant CpG site with LA, GA, and self-reported race, respectively, yielding the *p*-value cutoff of significant enrichment to be 5.75e–4 (0.05/87), 2.17e–3 (0.05/23), and 6.10e–4 (0.05/82).

### meQTL identification

Consistent with the LA EWAS, the meQTL identification was also performed in AA samples. We compared meQTLs identified by the following two models (Table 3) in order to identify meQTL that were and were not influenced by local ancestry. Local ancestry was adjusted in both models to control for the confounding effects from ancestry background. The first was a conventional model that identified the association between DNA methylation and genotypes regardless of the ancestral origin of the genotype. The p-value of the meQTL is derived from an *F* test comparing the conventional model to the null model. The second model allows the genetic effects to be different for SNPs with an African or European ancestry background (*b*_AFR_ and *b*_EUR_, respectively) and we further test the significance of the difference in a SNP’s effects by ancestry (*b*_diff_ = *b*_AFR _– *b*_EUR_). The *p*-value of the meQTL is derived from an *F* test comparing the ancestry model to the null model and the *p*-value of the SNP effects difference by ancestry (*b*_diff_) is derived from an *F* test comparing the ancestry model to the conventional model. The significance cutoff for meQTLs is *p*-value < 1.35e–8 for both models, which is based on applying a Bonferroni correction to the total number of DNA methylation-SNP pairs. The significance cutoff for the SNP effects difference by ancestry is *p*-value < 3.31e–7, again based on application of Bonferroni correction to the total number of meQTLs identified by the ancestry model.

As genetic variants are correlated due to linkage disequilibrium (LD), we performed clumping of the SNPs identified as meQTL either by the conventional or ancestry model. We defined a proxy independent locus as those featuring an LD r-square < 0.01. As LD blocks for AAs are relatively short, a locus with fewer than 10 SNPs or within 250 kb from another locus were merged into its nearest clump. The SNP with the lowest *p*-values in a clump was declared as the lead SNP. We used *p*-values from an *F* test against null models to identify lead SNPs for ancestry and conventional models, respectively. We examined the meQTLs in the VACS replication and WIHS groups, only considering DNA methylation associations replicated in the two groups in the EWAS stage. The significance threshold for the replication of meQTLs was set at *p*-value < 3.31e–5 for the VACS internal replication group and *p*-value < 1.04e–4 for the external WIHS replication group. The significance threshold for the replication of SNP effects difference by ancestry was set at *p*-value < 4.26e–5 for the VACS internal replication group and *p*-value < 1.19e–4 for the external WIHS replication group. R 4.0.3 was used for implementation of meQTL models.

### Reporting summary

Further information on research design is available in the Nature Research Reporting Summary linked to this article.

### Supplementary information

Supplementary Information

Description of Additional Supplementary Files

Supplementary Data 1.

Supplementary Data 2.

Supplementary Data 3.

Supplementary Data 4.

Supplementary Data 5.

Supplementary Data 6.

Supplementary Data 7.

Supplementary Data 8.

Supplementary Data 9.

Supplementary Data 10.

Supplementary Data 11.

Supplementary Data 12.

Supplementary Data 13.

Reporting Summary

## Data Availability

Demographic and clinical characteristics and DNA methylation data are submitted to the GEO dataset (GSE117861) and are available to the public. The source data of Fig. 4 are provided in Table 2 and Supplementary Data 4. The source data of Fig. 5 are provided in Supplementary Data 9-13. EWAS summary statistics are available at 10.6084/m9.figshare.19576264.v4.

## Code availability

All codes for analysis are also available upon a request to the corresponding author.
