# Supplementary material for: Incorporating local ancestry improves identification of ancestry-associated methylation signatures and meQTLs in African Americans
Source: Commun Biol. 2022 Apr 29;5:401. doi: 10.1038/s42003-022-03353-5 (PMC9054854; doi:10.1038/s42003-022-03353-5)
Supplement: Supplementary file 1 — Supplementary Information [file 42003_2022_3353_MOESM1_ESM.pdf]

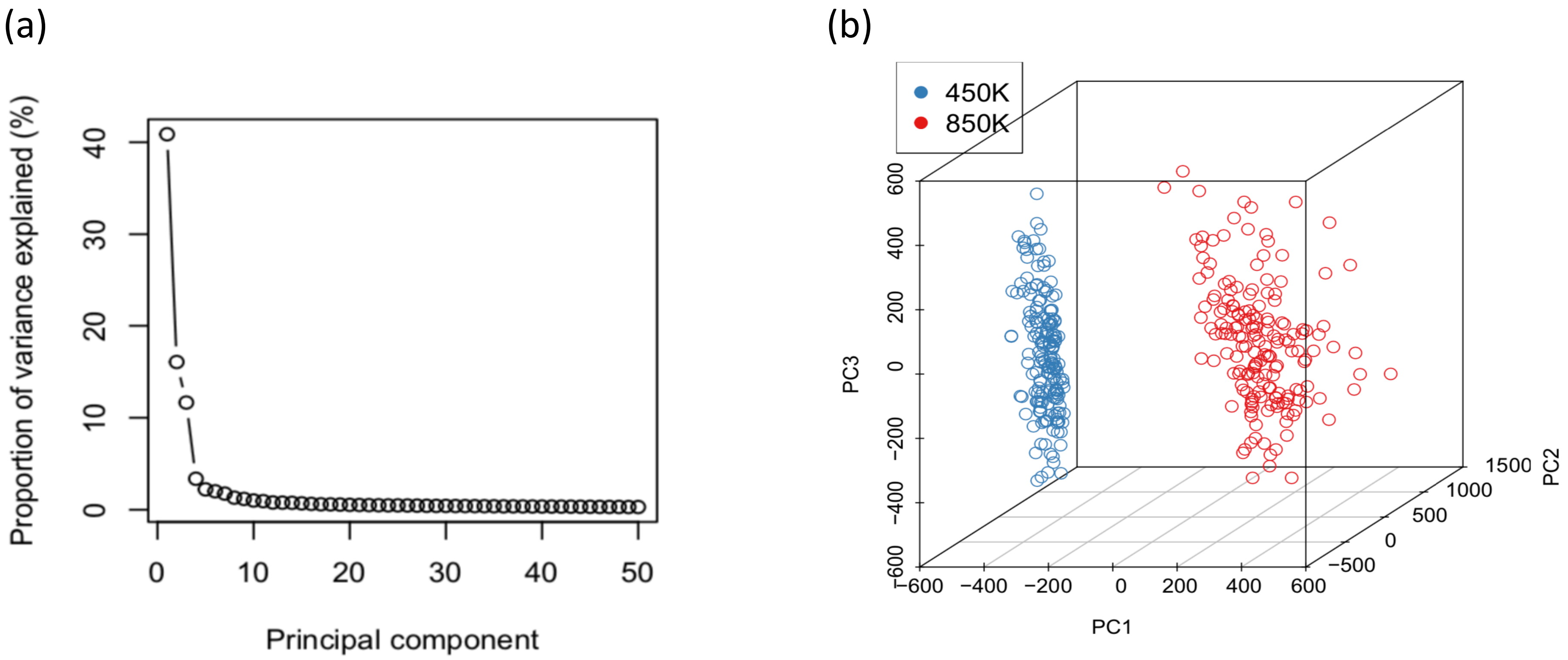

Supplementary Figure 1. Principal component analysis (PCA) of 408,583 common probes shared between HM450K and EPIC arrays in 176 samples who were measured with both arrays. (a) Variance explained by the top 50 PCs. (b) The top 3 PC plot showed a distinction between DNA methylation measured by two arrays at the common probes in same samples.

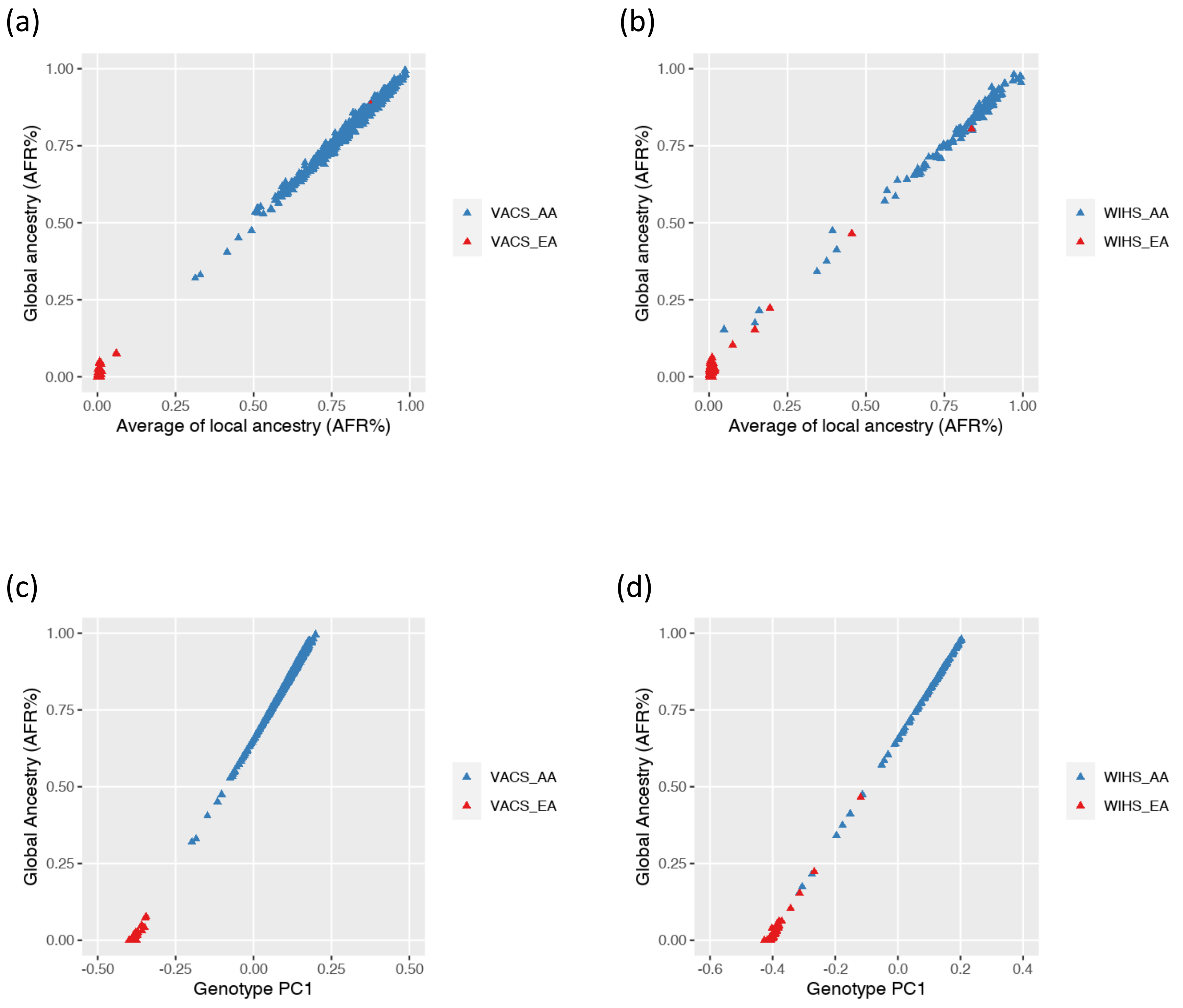

Supplementary Figure 2. Comparison of the global ancestry, the average of local ancestry, and genotype principal component (PC). The average of local African ancestry estimates is approximately identical to the global African ancestry estimate in (a) the Veterans Aging Cohort Study (VACS) samples (Pearson correlation=0.999, p-value<2e-16) and (b) the Women's Interagency HIV Study (WIHS) samples (Pearson correlation=0.999, p-value<2e-16). The global African ancestry is proportional to the first PC of genotypes for African Americans and European Americans in (c) the VACS (Pearson correlation=1, p-value<2e-16) and (d) the WIHS (Pearson correlation=1, p-value<2e-16).

(a)

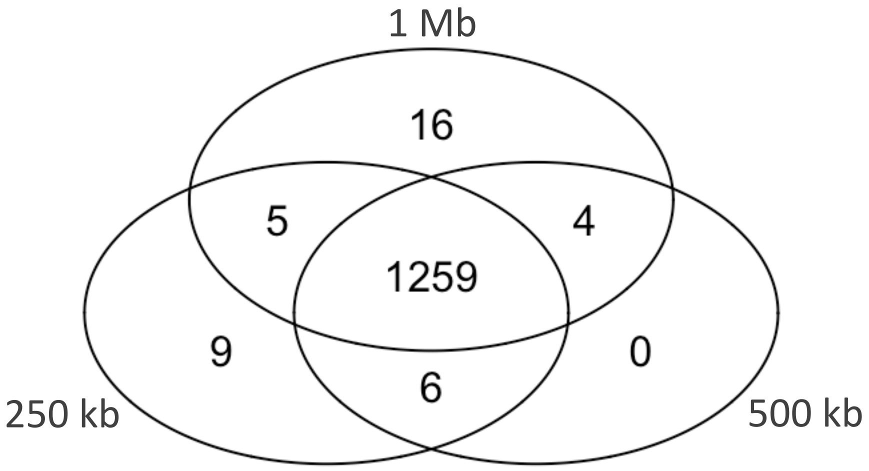

(b)

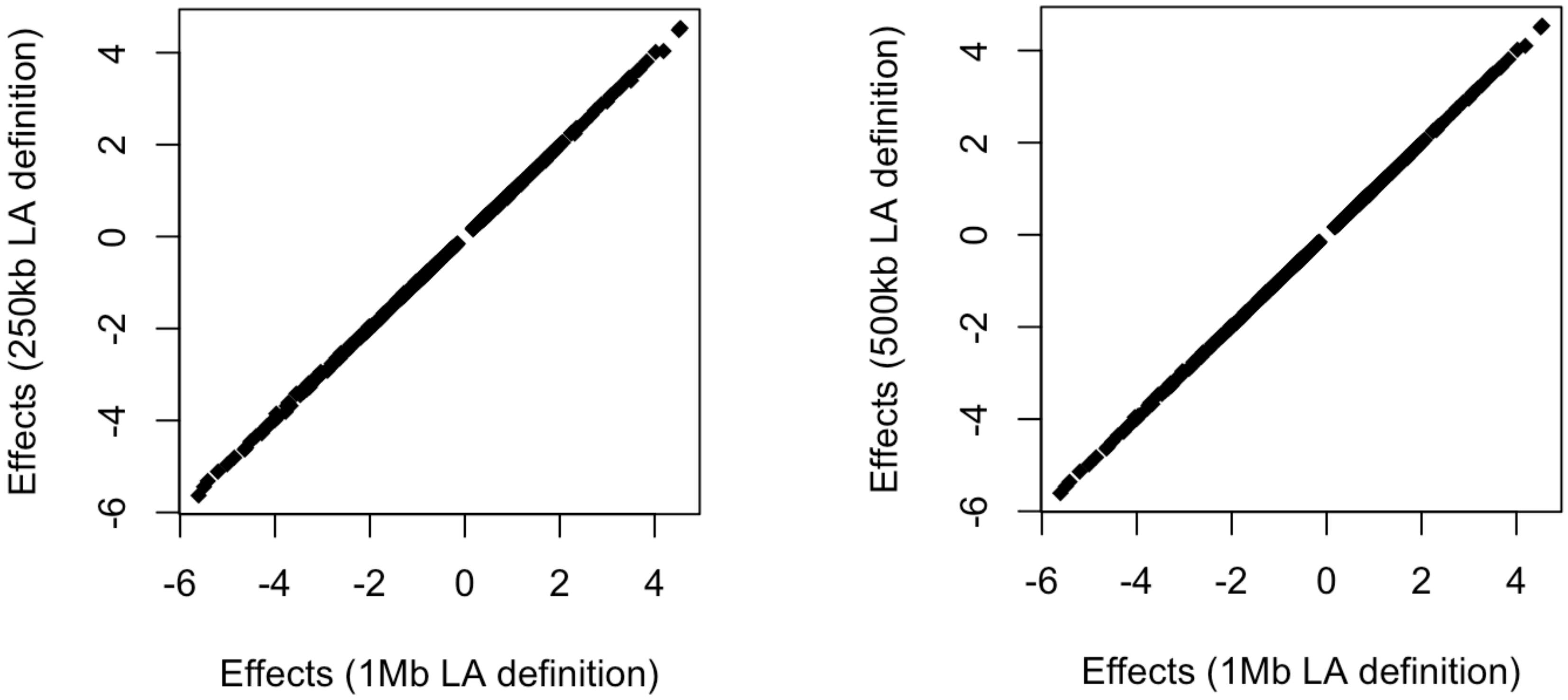

Supplementary Figure 3. **Sensitivity analysis of different flanking regions (250 kb, 500 kb, and 1 Mb) used in local ancestry (LA) definition on epigenome-wide association study (EWAS) association results.** (a) Venn plot of the identified EWAS associations using 250 kb, 500 kb, and 1 Mb LA definition. (b) The estimated effects were highly consistent among EWASs using different flanking region definitions (1 Mb vs. 250 kb: Pearson correlation>0.99, p-value<2.2e-16; 1 Mb vs. 500 kb: Pearson correlation>0.99, p-value<2.2e-16).

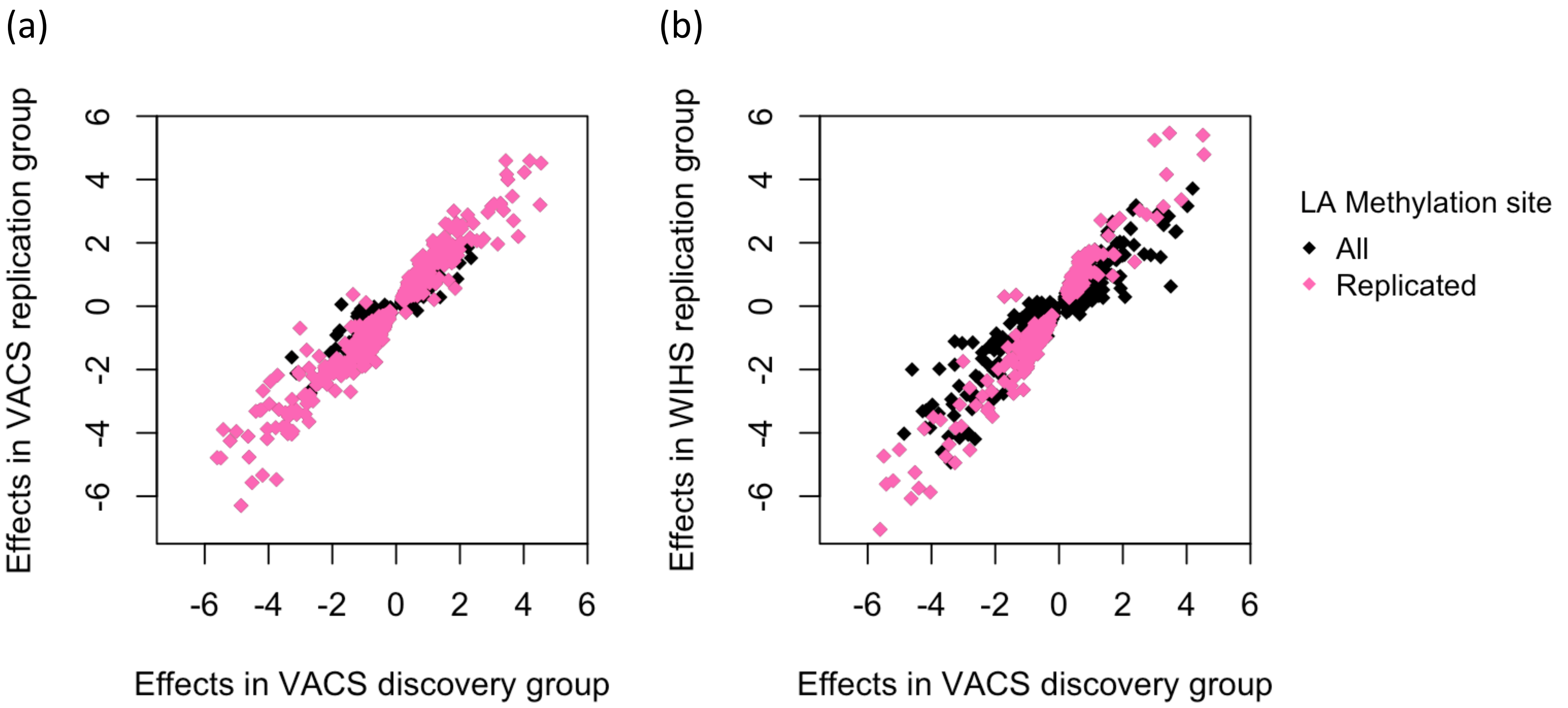

Supplementary Figure 4. **The identified significant effects of local ancestry on DNA methylation are highly correlated between the Veterans Aging Cohort Study (VACS) discovery group and both replication cohorts.** (a) 771 of 1,284 local ancestry associated methylation sites are replicated with concordant direction of effects in the VACS internal replication group. (b) 223 of 1,284 local ancestry associated methylation sites are replicated in the Women's Interagency HIV Study (WIHS) external replication group. Replicated associations (p-value<3.89e-5) are highlighted in pink.

Supplementary Note 1: The Two-Stage EWAS Model

We applied a two-stage EWAS model to control for technical and biological confounders and reduce EWAS inflation factor [1, 2]. First we constructed a model regressing the methylation M-value on all covariates (e.g., age, smoking status, cell-type composition, control probe PCs) excluding the ancestry variable of interest (self-reported race, GA, or LA) and obtained the PCs of the residuals. The top 5 residual PCs were then adjusted in the second-stage model to reduce the correlation between DNA methylation and test statistic inflation. In the second stage model, we regressed the methylation M-value on the ancestry variable of interest, the covariates included in the first model, and the top 5 residual PCs from the first model. Although DNA methylation beta-value has a more intuitive biological interpretation, the heteroscedasticity for highly methylated or unmethylated CpG sites (beta-value close to 1 and 0) is susceptible to violation of linear model assumptions [3]. Thus we used the approximately homoscedastic methylation M-value as response variable in both modeling stages for statistical validity.

We first performed a self-reported race epigenome-wide association study (EWAS) in European American (EA) and African American (AA) samples. Next, we conditioned on self-reported race and restrained the global and local ancestry-based EWAS in AA samples to pinpoint methylation signatures associated with genetic ancestry. We did not include EA samples in the EWAS of global and local ancestry because race can be a confounder in the EWAS and merely adjusting race as a covariate may not fully address the population structure. Finally in the local ancestry EWAS, we adjusted global ancestry as a covariate. In summary, no other ancestry variable was adjusted in the race EWAS. In the global ancestry EWAS, we restrained to AA samples (more stringent than adjusting for race). In the local ancestry EWAS, we restrained to AA samples and adjusted global ancestry.

Abbreviation
ADH: Adherence to medication
VL: Viral load
WBC: White blood cells
CD4: CD4 T cells
CD8: CD8 T cells
Granulocyte: Granulocytes
NK: Nature killer cells
Bcell: B cells
Monocyte: Monocytes
PCControlProbe: Control probe principal component
PCResidual: residual principal component
GA: Global ancestry
LA: Local ancestry

VACS self-reported race

Methylation M-value ~ age + smoker + log(PEth) + ADH + log(VL) + WBC + CD4 + CD8 + Granulocyte + NK + Bcell + Monocyte + PC1ControlProbe + … + PC30ControlProbe

Methylation M-value ~ self-reported race + age + smoker + log(PEth) + ADH + log(VL) + WBC + CD4 + CD8 + Granulocyte + NK + Bcell + Monocyte + PC1ControlProbe + … + PC30ControlProbe + PC1Residual + … + PC5Residual

VACS global ancestry

Methylation M-value ~ age + smoker + log(PEth) + ADH + log(VL) + WBC + CD4 + CD8 + Granulocyte + NK + Bcell + Monocyte + PC1ControlProbe + … + PC30ControlProbe

Methylation M-value ~ GA + age + smoker + log(PEth) + ADH + log(VL) + WBC + CD4 + CD8 + Granulocyte + NK + Bcell + Monocyte + PC1ControlProbe + … + PC30ControlProbe + PC1Residual + … + PC5Residual

VACS local ancestry

Methylation M-value ~ GA + age + smoker + log(PEth) + ADH + log(VL) + WBC + CD4 + CD8 + Granulocyte + NK + Bcell + Monocyte + PC1ControlProbe + … + PC30ControlProbe

Methylation M-value ~ LA + GA + age + smoker + log(PEth) + ADH + log(VL) + WBC + CD4 + CD8 + Granulocyte + NK + Bcell + Monocyte + PC1ControlProbe + … + PC30ControlProbe + PC1Residual + … + PC5Residual

WIHS self-reported race

Methylation M-value ~ age + smoker + hazardous drinker + HIV status + log(VL) + CD4 + CD8 + Granulocyte + NK + Bcell + Monocyte + PC1ControlProbe + … + PC10ControlProbe

Methylation M-value ~ self-reported race + age + smoker + hazardous drinker + HIV status + log(VL) + CD4 + CD8 + Granulocyte + NK + Bcell + Monocyte + PC1ControlProbe + … + PC10ControlProbe + PC1Residual + … + PC5Residual

WIHS global ancestry

Methylation M-value ~ age + smoker + hazardous drinker + HIV status + log(VL) + CD4 + CD8 + Granulocyte + NK + Bcell + Monocyte + PC1ControlProbe + … + PC10ControlProbe

Methylation M-value ~ GA + age + smoker + hazardous drinker + HIV status + log(VL) + CD4 + CD8 + Granulocyte + NK + Bcell + Monocyte + PC1ControlProbe + … + PC10ControlProbe + PC1Residual + … + PC5Residual

WIHS local ancestry

Methylation M-value ~ GA + age + smoker + hazardous drinker + HIV status + log(VL) + CD4 + CD8 + Granulocyte + NK + Bcell + Monocyte + PC1ControlProbe + … + PC10ControlProbe

Methylation M-value ~ LA + GA + age + smoker + hazardous drinker + HIV status + log(VL) + CD4 + CD8 + Granulocyte + NK + Bcell + Monocyte + PC1ControlProbe + … + PC10ControlProbe + PC1Residual + … + PC5Residual

Supplementary References

1. Lehne B, *et al.* A coherent approach for analysis of the Illumina HumanMethylation450 BeadChip improves data quality and performance in epigenome-wide association studies. *Genome Biology* **16**, 37 (2015).

2. Zhang X, *et al.* DNA methylation signatures of illicit drug injection and hepatitis C are associated with HIV frailty. *Nature Communications* **8**, 2243 (2017).

3. Du P, *et al.* Comparison of Beta-value and M-value methods for quantifying methylation levels by microarray analysis. *BMC Bioinformatics* **11**, 587 (2010).
