# Supplementary material for: Incorporating local ancestry improves identification of ancestry-associated methylation signatures and meQTLs in African Americans
Source: Commun Biol. 2022 Apr 29;5:401. doi: 10.1038/s42003-022-03353-5 (PMC9054854; doi:10.1038/s42003-022-03353-5)
Supplement: Supplementary file 2 — Description of Additional Supplementary Files [file 42003_2022_3353_MOESM2_ESM.pdf]

Description of Additional Supplementary Files

File name: Supplementary Data 1
Description: Epigenome-wide significant methylation sites (p-value<1.16e-7) associated with self-reported race in the Veterans Aging Cohort Study (VACS) discovery group (N=527) and replications in the VACS replication group (N=467) and Women's Interagency HIV Study (WIHS) replication cohort (N=230)

File name: Supplementary Data 2
Description: Epigenome-wide significant methylation sites (p-value<1.16e-7) associated with global ancestry in the Veterans Aging Cohort Study (VACS) discovery group (N=478) and replications in the VACS replication group (N=422) and Women's Interagency HIV Study (WIHS) replication cohort (N=131)

File name: Supplementary Data 3
Description: Epigenome-wide significant methylation sites (p-value<1.16e-7) associated with local ancestry in the Veterans Aging Cohort Study (VACS) discovery group (N=478) and replications in the VACS replication group (N=422) and Women's Interagency HIV Study (WIHS) replication cohort (N=131)

File name: Supplementary Data 4
Description: The SNP-based heritability estimates of local ancestry-associated DNA methylation (mean h2=0.45, median h2=0.43) and the overall heritability (mean h2=0.06, median h2=0.01) estimated from all methylation sites

File name: Supplementary Data 5
Description: The trait enrichment of the differentially methylated CpG sites identified in the EWAS of local ancestry, global ancestry, and self-reported race

File name: Supplementary Data 6
Description: Methylation quantitative trait loci (meQTL) identified from the conventional model for local ancestry associated DNA methylation in the Veterans Aging Cohort Study (VACS) discovery group (N=478)

File name: Supplementary Data 7
Description: Methylation quantitative trait loci (meQTL) identified from the ancestry model for local ancestry associated DNA methylation in the Veterans Aging Cohort Study (VACS) discovery group (N=478), and replications in the VACS replication group (N=422) and Women's Interagency HIV Study (WIHS) replication cohort (N=131)

File name: Supplementary Data 8
Description: Methylation quantitative trait loci (meQTL) with genetic effects significantly differed by ancestry for local ancestry associated DNA methylation in the Veterans Aging Cohort Study (VACS) discovery group (N=478), and replications in the VACS replication group (N=422) and Women's Interagency HIV Study (WIHS) replication cohort (N=131)

File name: Supplementary Data 9
Description: meQTL rs9370878 of CpG site cg20133046: the genetic effects on the methylations are opposite for the African and European ancestry

File name: Supplementary Data 10
Description: meQTL rs1552489 of CpG site cg08033130: the genetic effects from the two ancestries contribute to the methylation in the same direction but with different effect sizes

File name: Supplementary Data 11
Description: meQTL rs2955229 of CpG site cg24599650: the African alleles are associated with differential methylations while the European alleles are not

File name: Supplementary Data 12
Description: meQTL rs4074196 of CpG site cg23611477: the European alleles are associated with differential methylations while the African alleles are not

File name: Supplementary Data 13
Description: meQTL rs6774733 of CpG site cg02527881: the genetic effects are comparable between ancestries
