# Supplementary material for: Incorporating local ancestry improves identification of ancestry-associated methylation signatures and meQTLs in African Americans
Source: Commun Biol. 2022 Apr 29;5:401. doi: 10.1038/s42003-022-03353-5 (PMC9054854; doi:10.1038/s42003-022-03353-5)
Supplement: Supplementary file 16 — Reporting Summary [file 42003_2022_3353_MOESM16_ESM.pdf]

nature portfolio

Corresponding author(s): Dr. Hongyu Zhao and Dr. Ke Xu

Last updated by author(s): Apr 7, 2022

Reporting Summary

Nature Portfolio wishes to improve the reproducibility of the work that we publish. This form provides structure for consistency and transparency in reporting. For further information on Nature Portfolio policies, see our Editorial Policies and the Editorial Policy Checklist.

Statistics

For all statistical analyses, confirm that the following items are present in the figure legend, table legend, main text, or Methods section.

n/a	Confirmed	
☐	☒	The exact sample size (*n*) for each experimental group/condition, given as a discrete number and unit of measurement
☐	☒	A statement on whether measurements were taken from distinct samples or whether the same sample was measured repeatedly
☐	☒	The statistical test(s) used AND whether they are one- or two-sided<br>*Only common tests should be described solely by name; describe more complex techniques in the Methods section.*
☐	☒	A description of all covariates tested
☐	☒	A description of any assumptions or corrections, such as tests of normality and adjustment for multiple comparisons
☐	☒	A full description of the statistical parameters including central tendency (e.g. means) or other basic estimates (e.g. regression coefficient) AND variation (e.g. standard deviation) or associated estimates of uncertainty (e.g. confidence intervals)
☐	☒	For null hypothesis testing, the test statistic (e.g. *F*, *t*, *r*) with confidence intervals, effect sizes, degrees of freedom and *P* value noted<br>*Give P values as exact values whenever suitable.*
☒	☐	For Bayesian analysis, information on the choice of priors and Markov chain Monte Carlo settings
☒	☐	For hierarchical and complex designs, identification of the appropriate level for tests and full reporting of outcomes
☐	☒	Estimates of effect sizes (e.g. Cohen's *d*, Pearson's *r*), indicating how they were calculated

Our web collection on statistics for biologists contains articles on many of the points above.

Software and code

Policy information about availability of computer code

Data collection	The Illumina Infinium HumanMethylation450 BeadChip (HM450K) and the Illumina Infinium MethylationEPIC BeadChip (EPIC) were used for DNA methylation profiling of study participants. The VACS samples were genotyped using the Illumina HumanOmniExpress Beadchip and imputation was performed using IMUPTE2. The WIHS samples were genotyped using the Infinium Omni2.5 BeadChip and Minimac4 for imputation.
Data analysis	PLINK 1.9 was used for genotype quality control and genetic variants pruning. ADMIXTURE 1.3.0 was used for global ancestry estimation. SHAPEIT2 was used for phasing genotype data. RFMix 1.5.4 was used for local ancestry inference. GCTA 1.93.2 was used for SNP-based heritability estimation. EWAS Atlas database was used for trait enrichment analysis. R 4.0.3 was used for EWAS and meQTL analysis and data visualization.

For manuscripts utilizing custom algorithms or software that are central to the research but not yet described in published literature, software must be made available to editors and reviewers. We strongly encourage code deposition in a community repository (e.g. GitHub). See the Nature Portfolio guidelines for submitting code & software for further information.

Data

Policy information about availability of data

All manuscripts must include a data availability statement. This statement should provide the following information, where applicable:

- Accession codes, unique identifiers, or web links for publicly available datasets
- A description of any restrictions on data availability
- For clinical datasets or third party data, please ensure that the statement adheres to our policy

Demographic and clinical characteristics and DNA methylation data for the VACS samples are submitted to the GEO dataset (GSE117861) and are available to the

public. The source data of Figure 4 are provided in Table2 and Supplementary Data 4. The source data of Figure 5 are provided in Supplementary Data 9-14. EWAS summary statistics are available at https://yale.box.com/s/fist5zdioui6bvfj9oshjekd6lmtgej7.

Field-specific reporting

Please select the one below that is the best fit for your research. If you are not sure, read the appropriate sections before making your selection.

☒ Life sciences ☐ Behavioural & social sciences ☐ Ecological, evolutionary & environmental sciences

For a reference copy of the document with all sections, see nature.com/documents/nr-reporting-summary-flat.pdf

Life sciences study design

All studies must disclose on these points even when the disclosure is negative.

Sample size	We included all African American (AA) and European American (EA) samples from VACS (N=994) and WIHS (N=230) whose DNA methylation and genotype data were both available.
Data exclusions	We focused on AA and EA samples in this study. Participants whose self-reported race were other than these two were excluded. We restricted the analysis to samples from HIV-positive male participants in VACS to eliminate the effects of HIV status and sex on the methylation level in the discovery and internal replication analyses. This criterion resulted in limited sample loss since the majority of VACS participants were males and tested positive for HIV.
Replication	The VACS EPIC samples served as an internal replication group (N_AA=422, N_EA=45). The WIHS cohort (N_AA=131, N_EA=99) served as an external replication cohort. The two groups were used for replication of EWAS and meQTL findings. We summarized the replication results in Supplementary Table 1-3, 6.
Randomization	The VACS and WIHS are prospective, observational cohort studies. Randomization is not applicable.
Blinding	Genotyping was done blind to phenotypes.

Reporting for specific materials, systems and methods

We require information from authors about some types of materials, experimental systems and methods used in many studies. Here, indicate whether each material, system or method listed is relevant to your study. If you are not sure if a list item applies to your research, read the appropriate section before selecting a response.

Materials & experimental systems

n/a	Involved in the study	
☒	☐	Antibodies
☒	☐	Eukaryotic cell lines
☒	☐	Palaeontology and archaeology
☒	☐	Animals and other organisms
☐	☒	Human research participants
☒	☐	Clinical data
☒	☐	Dual use research of concern

Methods

n/a	Involved in the study	
☒	☐	ChIP-seq
☒	☐	Flow cytometry
☒	☐	MRI-based neuroimaging

Human research participants

Policy information about studies involving human research participants

Population characteristics	After applying the inclusion and exclusion criteria, the VACS discovery group were 100% HIV-positive males, including 478 AA and 49 EA samples. The mean age for AA and EA samples were 49.44 and 49.04 years. respectively. In the VACS internal replication group, samples were also 100% HIV-positive males, including 422 AA and 45 EA samples. The mean age for AA and EA samples were 47.88 and 48.22 years. respectively. In WIHS, samples were 100% females, including 131 AA (64% HIV-positive) and 99 EA (54% HIV-positive) samples. The mean age for AA and EA samples were 44.04 and 44.69 years. respectively. Demographic and clinical characteristics for the three groups are summarized in Table 1.
Recruitment	The VACS and the WIHS are both multi-center, prospective, observational cohort studies. The VACS recruited HIV-positive cases and age-, race-, site-matched HIV-negative controls where the majority of participants are men. In WIHS, all participants are women infected with HIV or at risk for HIV acquisition. We studied DNA methylation and genetic data measured in samples contributed by of AA and EA participants from the two cohorts.
Ethics oversight	The study was approved by the central Veteran Affairs Institutional Review Board (IRB) at participating sites. All relevant ethical regulations for work with human subjects were followed in the conduct of the study and informed consent was obtained from all participants.

Note that full information on the approval of the study protocol must also be provided in the manuscript.
